# Influence of Magnesium Degradation on Schwannoma Cell Responses to Nerve Injury Using an In Vitro Injury Model

**DOI:** 10.3390/jfb15040088

**Published:** 2024-03-31

**Authors:** Krathika Bhat, Lisa Hanke, Heike Helmholz, Eckhard Quandt, Sarah Pixley, Regine Willumeit-Römer

**Affiliations:** 1 Institute of Metallic Biomaterials, Helmholtz Zentrum Hereon, 21502 Geesthacht, Germany; 2 Institute of Materials Science, University of Kiel, 24143 Kiel, Germany; 3 College of Medicine, University of Cincinnati, Cincinnati, OH 45267-0576, USA

**Keywords:** magnesium, lithium, biodegradable, Schwann cells, nerve injury, in vitro

## Abstract

Nerve guidance conduits for peripheral nerve injuries can be improved using bioactive materials such as magnesium (Mg) and its alloys, which could provide both structural and trophic support. Therefore, we investigated whether exposure to Mg and Mg-1.6wt%Li thin films (Mg/Mg-1.6Li) would alter acute Schwann cell responses to injury. Using the RT4-D6P2T Schwannoma cell line (SCs), we tested extracts from freeze-killed cells (FKC) and nerves (FKN) as in vitro injury stimulants. Both FKC and FKN induced SC release of the macrophage chemoattractant protein 1 (MCP-1), a marker of the repair SC phenotype after injury. Next, FKC-stimulated cells exposed to Mg/Mg-1.6Li reduced MCP-1 release by 30%, suggesting that these materials could have anti-inflammatory effects. Exposing FKC-treated cells to Mg/Mg-1.6Li reduced the gene expression of the nerve growth factor (NGF), glial cell line-derived neurotrophic factor (GDNF), and myelin protein zero (MPZ), but not the p75 neurotrophin receptor. In the absence of FKC, Mg/Mg-1.6Li treatment increased the expression of NGF, p75, and MPZ, which can be beneficial to nerve regeneration. Thus, the presence of Mg can differentially alter SCs, depending on the microenvironment. These results demonstrate the applicability of this in vitro nerve injury model, and that Mg has wide-ranging effects on the repair SC phenotype.

## 1. Introduction

Peripheral nerve injuries resulting in long nerve gaps require support to facilitate complete axonal regeneration. While autografts are the current gold standard for this purpose, the associated challenges such as donor site lesion and limited quantity have prompted extensive research into the development of nerve guidance conduits [1,2]. Hollow nerve conduits, some of which are in current clinical use, serve to mechanically bridge the gap in the injured nerve and enclose the severed nerve to create a regenerative environment by allowing the enrichment of growth factors and preventing fibrosis and inflammation [1]. However, there remains a need to improve the functionality of such conduits by using bioactive materials, delivering additional growth factors, and adding micropatterns for further support and guided growth [1,3]. 

Upon nerve injury, Schwann cells (SCs) play many key roles in facilitating regeneration, including macrophage recruitment, phagocytosis of axonal and myelin debris (collectively termed Wallerian degeneration), proliferation, migration, promotion and guidance of axon regeneration and remyelination. To accomplish these roles, SCs undergo dedifferentiation (transdifferentiation) after axonal injury into a repair phenotype. Repair SC actions include, among other effects, the downregulation of myelin protein secretion, the upregulation of pro-inflammatory factors such as macrophage chemoattractant protein 1 (MCP-1) and tumor necrosis factor α (TNFα), and an increase in the secretion of growth factors such as nerve growth factor (NGF), glial cell line-derived neurotrophic factor (GDNF), and brain-derived neurotrophic factor (BDNF) [4,5]. Due to the indispensable roles of these factors in nerve regeneration, it is important to understand the influence of biomaterials used in nerve regeneration on SC function.

Magnesium (Mg)-based materials could be candidate materials for addition to conduits or to provide physical contact guidance inside a conduit. Mg implants are being used at the clinical level in orthopedic, dental, and cardiovascular applications, for both the mechanical properties of the Mg metal and the biological and the anti-inflammatory effects of the released Mg ions [6,7,8]. Nervous tissue and neurological applications have focused more on the biological rather than the mechanical properties, as only minimal strength is required for these applications [9,10]. Preliminary research from the last decade, as outlined below, suggests that Mg could be beneficial to nerve healing. Mg is biocompatible, biodegradable, and mechanically stable enough to support nerve regrowth [11]. Moreover, Mg, as a metal, offers electrical conductance [11], and conductive materials can promote nerve regeneration [12]. Mg ions are known to have a neuroprotective effect by serving as an antagonist to the N-methyl-D-aspartic acid glutamate receptor and calcium ions. The release of glutamate and calcium after axonal injury can cause apoptosis in neighboring cells, and Mg has been shown to prevent this [13]. Mg has also been shown to have anti-inflammatory effects [13], and all these effects are beneficial to nerve repair [11]. Several in vitro and in vivo studies have shown that Mg improves nerve regeneration through reduced inflammation [14], enhanced remyelination and growth factor expression [15,16,17], improved neurite outgrowth [16,17,18,19], axon number [20], and improved functional recovery [15]. In particular, Li et al. and Pixley et al. have used Mg metal wires in their studies, with positive effects on nerve repair [15,20,21,22]. However, while many of the in vivo studies have targeted the effects on axons and inflammation, observing the nerve as a whole, few studies have specifically studied the effects on SCs [14,23], and the in vitro studies focused mainly on cytocompatibility [24,25] or did not consider the injury microenvironment [17,19]. In summary, there is a need for improved understanding of SC responses to Mg material degradation.

The effects of metallic Mg materials on nervous tissue repair could be improved by using alloying elements such as lithium (Li). Li is widely used to treat bipolar disorder, and it exerts several neuroprotective effects, such as upregulating anti-apoptotic factors and growth factors and reducing inflammation [26,27]. Li administration has promoted nerve regeneration in vivo by alleviating inflammation [28,29], enhancing autophagy [30,31,32,33], improving remyelination [30,34,35] and improving functional recovery [36]. Some mechanisms of action of Li are attributed to its inhibition of the enzymes glycogen synthase kinase-3β (GSK3β) and inositol phosphate phosphatase. The enzyme GSK3β has been shown to influence SC functions such as autophagy and remyelination during nerve regeneration. Therefore, Li, an inhibitor of this enzyme, can influence nerve regeneration via its actions on GSK3β [23,30,37]. We propose that using Li as an alloying element combined with Mg metal could enhance the beneficial effects on nerve regeneration. Sun et al. published a nerve regeneration study using Li-Mg-Si bioceramics, demonstrating beneficial effects both in vitro and in vivo [23]. They found that the material promoted the myelinating phenotype of SCs in a GSK3β/β-catenin dependent manner, suggesting that Mg-Li materials show promise for use in nerve regeneration.

We studied the effects of biodegradable Mg and Mg-1.6wt%Li metal thin films (Mg/Mg-1.6Li) on the acute responses of the RT4-D6P2T Schwannoma cell line. The Li content in the alloy was chosen to deliver lithium concentrations close to therapeutically relevant levels upon degradation [38]. SC exposure to reparative metals would occur under injury conditions, and would either be implanted right after injury or later during surgical repair and insertion. Therefore, to understand the cellular responses in an injury-like environment, we first tested two in vitro nerve injury models—freeze-killed cell extract (FKC) and nerve extract (FKN)—by using MCP-1 release as a marker. To our knowledge, this is the first study to use these injury models with the RT4-D6P2T cell line, a widely used cell line employed in peripheral nervous system studies, evaluate their effects on cellular responses to Mg and Li. Using the FKC extract to simulate injury conditions, we further tested the effect of Mg/Mg-1.6Li materials on chemokines, myelin, and neurotrophin gene expression in RT4-D6P2T cells in order to simulate adding a metal implant to aid recovery. This study improves our understanding of the effects of the degradation products of Mg, with and without Li, on SC responses relevant to nerve regeneration. 

## 2. Materials and Methods

### 2.1. Fabrication and Cleaning of Mg and Mg-1.6Li Thin Films for Cell Culture

Freestanding Mg and Mg-1.6wt%Li (Mg-1.6Li) thin films were fabricated by UV lithography and magnetron sputtering at CAU Kiel. The fabrication process and material characterization for these types of thin films have already been published in detail [39]. In brief, a photoresist coating was applied on a silicon wafer (4″ diameter), and a mask aligner (MA6/BA6, Süss MicroTec, Garching, Germany) was used to create the pattern for 5 × 5 mm thin films. The wafer was then coated with aluminum (Al), which served as a hard mask, after photoresist lift-off. After etching (ICP-RIE SI 500, SenTech, Berlin, Germany), a sacrificial layer of aluminum nitride (AlN) was sputtered onto the surface, followed by an Mg or Mg-1.6Li layer of 20 µm thickness. The Mg-1.6Li sputtering target was composed of Mg-2.5wt%Li (FHR, Ottendorf-Okrilla, Germany). The Mg or Mg-1.6Li thin films were released by etching away the sacrificial Al/AlN layer using 20% potassium hydroxide. For cell culture, the thin films were cleaned by submerging them in n-hexane and acetone for 20 min each. Then, they were cleaned in pure ethanol for 3 min and stored in vacuum-sealed bags until use. Shortly before their use for cell culture, the thin films were sterilized by submerging them in 70% ethanol for 20 min and allowed to dry in a sterile atmosphere. Once dry, each thin film was placed inside a 24-well plate insert (0.4 µm pore size, 83.3932.040, Sarstedt, Nümbrecht, Germany).

### 2.2. Cell Culture

The RT4-D6P2T Schwannoma cell line was purchased from ATCC (CRL-2768, American Type Culture Collection, Manassas, VA, USA) and cultured in a humidified incubator (37 °C, 5% CO_2_, 95% humidity). For routine passaging, the cells were cultured in Dulbecco’s Modified Eagle’s Medium (DMEM) (10-013-CV, Corning, Somerville, MA, USA) supplemented with 10% fetal bovine serum (FBS) (10437010, Thermo Fisher Scientific, Carlsbad, CA, USA) and an antibiotic-antimycotic solution (1× AA, 091674049, MP Biomedicals, Santa Ana, CA, USA) (henceforth called growth medium). For the experiments, the cells were plated at a density of 20,000 cells in 1 mL/well in 24-well plates (3524, Corning, USA) or 2000 cells in 100 µL/well in 96-well plates (3595, Corning, USA) in growth medium. After 10 h, the medium was switched to DMEM supplemented with N2 (17502048, Thermo Fisher Scientific, USA) and 1× AA (henceforth called N2 medium).

### 2.3. MTT Assay 

To check the biocompatibility of the Mg and Mg-1.6Li thin films with the RT4-D6P2T cells, a 3-(4,5-dimethylthiazol)-2,5-diphenyltetrazolium bromide (MTT) assay was performed. The cellular metabolic activity determined from the assay was used as a measure of cell proliferation. After incubating the cells in N2 medium for 14 h in a 24-well plate, the inserts with the thin films were added to the wells. The cells with the N2 medium were used as the negative control. After an incubation period of 48 h, the inserts were removed, and the cells were washed with PBS. Then, 1 mL of 0.5 mg/mL MTT (M2128, Sigma-Aldrich, St. Louis, MO, USA) in growth medium was added to the wells and incubated for 3 h. The MTT solution was replaced with 400 µL of DMSO for the dissolution of formazan, and 100 µL of the formazan solution was transferred to a 96-well plate for measurement. The absorbance was read at 570 nm, and a reference wavelength of 660 nm was used (BioTek Cytation 5, Agilent, Santa Clara, CA, USA). The absorbance measurements were normalized to those of the untreated control. 

### 2.4. Harvesting Rat Sciatic Nerves

Two adult (male) Sprague Dawley rats were euthanized using methods consistent with the American Veterinary Medical Association Guidelines for the Euthanasia of Animals and approved by the University of Cincinnati Institutional Animal and Use Committee (UC-IACUC). The hind limbs were removed and rinsed in 70% ethanol. After removing the skin under a sterile atmosphere, the limbs were rinsed again in 70% ethanol, and the sciatic nerves from the sciatic notch to the distal entry into the muscles were removed. The nerves were cut into tiny pieces (~1 mm) using scalpels and transferred to a pre-weighed, sterile cryovial. The vial was weighed again to obtain the weight of harvested wet tissue and then frozen at −80 °C until preparation of the extracts.

### 2.5. Preparation of Freeze-Killed Cells and Nerve Extracts

Necrotic Schwann cell extract and rat nerve extract produced by freeze-killing were used as in vitro injury stimulants. The stimulants and preparation methods were based on the works of Lee et al. [40] and Karanth et al. [41]. RT4-D6P2T cells (10 × 10^6^ cells/mL) and nerve segments (100 mg wet tissue/mL) in cryovials, both in supplement-free DMEM, were subjected to five freeze-thaw cycles using a dry ice-ethanol slurry and a water bath (37 °C). Then, the vials were centrifuged at 13,000 rpm and 4 °C for 10 min. The supernatant was transferred to a new vial and stored at −80 °C until use. Before the experiment, FKC and FKN were mixed with N2 and 1× AA, diluted to the required dose in N2 medium, sterile filtered and used immediately.

### 2.6. Treatment of Cells with Injury Stimulants and Thin Films

The cells were seeded in 96-well plates in growth medium as described above, followed by incubation in N2 medium for 14 h. The injury stimulants FKC and FKN were then added at different doses to the wells, and the MCP-1 release was used as a marker to compare the two injury stimulants, as this chemokine is released from Schwann cells exposed to nerve injury [4]. Since this response is closely linked to toll-like receptor (TLR) signaling [42], TLR4 ligand bacterial lipopolysaccharide (LPS, L3024, Sigma-Aldrich, USA) was used as a positive control (1 µg/mL final concentration). Supernatant samples were collected at 24 h and 48 h for MCP-1 determination by ELISA (one plate for each time point). 

To study the effect of Mg/Mg-1.6Li thin film degradation on injury-stimulated Schwann cells, the RT4-D6P2T cells were seeded in 24-well plates, as described in Section 2.2, and incubated with FKC (5 × 10^6^ cells/mL dosage) and/or thin films for 48 h in N2 medium. SC cells were also exposed to the thin films without FKC to identify the effect of the material degradation in the later stages of healing, when the damage-associated factors would be mostly cleared from the SC microenvironment. The supernatant was sampled at 24 h intervals and stored at −80 °C for ELISA. Additionally, part of the supernatant was acidified to 1% HNO_3_ and stored at 4 °C for analysis by inductively coupled plasma optical emission spectroscopy (ICP-OES). After 48 h, the media was replaced with 300 µL of RNAlater^TM^ (AM7020, Thermo Fisher Scientific, Darmstadt, Germany), and the cells were scraped into the solution and stored at −20 °C for gene expression analysis.

### 2.7. Quantification of MCP-1 Release

The MCP-1 in the supernatant was quantified using a Rat JE/MCP-1/CCL2 DuoSet ELISA (DY3144, R&D systems, Minneapolis, MN, USA), as per the manufacturer’s protocol, using the ancillary reagents (DY008, R&D systems, USA). The absorbance was measured using a detection wavelength of 450 nm and a reference wavelength of 570 nm (BioTek Cytation 5, Agilent, USA).

### 2.8. Determination of Mg and Li Concentrations

The acidified supernatants were further diluted with 4% HNO_3_ in metal-free tubes (XX96.1, Carl Roth, Karlsruhe, Germany). A dilution factor of 1:100 to 1:1000 was employed, depending on the element concentration, and the final sample volume was 10 mL. The concentrations of Mg and Li were quantified using ICP-OES (SPECTROS-ARCOS, SPECTRO Analytical Instruments GmbH, Kleve, Germany). The wavelength used for Li was 670.8 nm and that of Mg was 280.3 nm. A calibration curve ranging from 0.05 to 2 mg/L was used for quantification. A certified multielement standard (33595.000 Bernd Kraft, AnalytiChem GmbH, Duisburg, Germany) was applied for the element determination.

### 2.9. RNA Extraction and Quantitative RT-PCR

The RNA from the cells stored in RNAlater^TM^ was extracted using a Quick-RNA Microprep Kit (R1051, Zymo Research, Freiburg, Germany), according to the manufacturer’s protocol. The RNA was eluted in 13 µL nuclease-free water, and the concentration was measured using the NanoDrop^TM^ 2000 (Thermo Scientific, Germany) by spectrophotometry. The RNA was used for cDNA synthesis using the RevertAid RT Kit (K1691, Thermo Fisher Scientific, Germany). Primers were designed using NCBI Primer-BLAST purchased from Eurofins Genomics (Ebersberg bei München, Germany). The primer list is provided in Table 1. qPCR was performed using PerfeCTa SYBR^®^ Green FastMix (95072, Quantabio, VWR, Darmstadt, Germany) and the CFX Opus 96 real-time PCR detection system (Bio-Rad, Feldkirchen, Germany). The input cDNA corresponded to 1 ng of total RNA, and the reaction involved an initial denaturation/activation step at 95 °C for 3 min, followed by 40 cycles of 95 °C for 20 s and 60 °C for 50 s. A melt curve analysis was performed at the end to confirm the melting temperature of the PCR product. The cycle threshold (Ct) values were determined from the software output (CFX Maestro, Bio-Rad, Germany) and were used to calculate the fold changes in gene expression using the ΔΔCt method [43]:

∆Ct_gene_ = Ct_gene_ − Ct_reference gene_

ΔΔCt_gene_ = ∆Ct_gene, test condition_ − ∆Ct_gene, control_

Fold change = 2^−ΔΔCt^

### 2.10. Statistical Analyses

Statistical analyses of the results were performed using GraphPad Prism 9.5.0. Analyses were performed on *n* = 6–9 data points obtained from 2–3 independent runs, with three technical replicates each. The outliers were excluded using Grubb’s test, and the normality of the data distribution was verified by the Shapiro–Wilk test. All results have been depicted as mean ± standard deviation. Significant differences between the groups were evaluated by *t*-test and one-way or two-way ANOVA, followed by Sidak’s post hoc test. Differences were considered to be statistically significant for values of *p* < 0.05.

## 3. Results

### 3.1. Mg-Based Extracts Showed Minimal Cytotoxicity with RT4-D6P2T Cells after Short-Term Exposure

**Figure 1:**
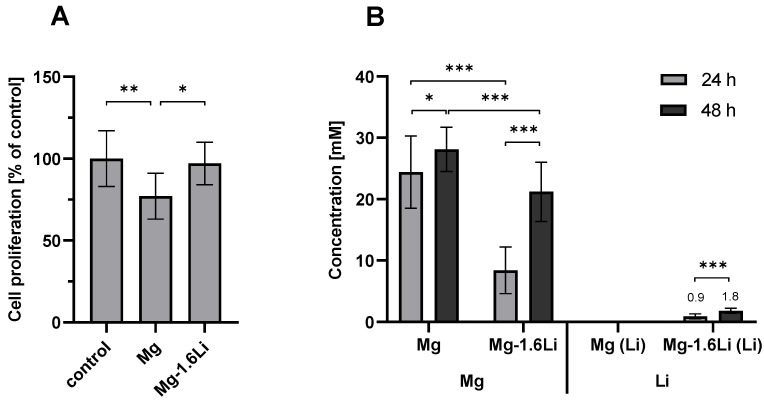
(**A**) Effect of Mg and Mg-1.6Li thin film degradation on RT4-D6P2T cell proliferation. The cell proliferation was measured by an MTT assay and is depicted relative to the untreated control. Significant differences were analyzed using one-way ANOVA (*p* = 0.0057). (**B**) Concentrations of Mg and Li in the supernatant resulting from Mg and Mg-1.6Li thin film degradation. The concentrations were measured by ICP-OES. Significant differences were analyzed using two-way ANOVA (1B, Mg concentrations, *p* < 0.001) or a *t*-test (1B, Li concentrations (*p* < 0.001). Following the ANOVAs, significance in the Sidak’s post hoc tests is indicated at *p* < 0.05 (*), *p* < 0.01 (**), *p* < 0.001 (***). Graphs show the mean ± SD of *n* = 9 data points (three replicates from three independent experiments).

The results from the MTT assay performed on the RT4-D6P2T cells indirectly exposed to Mg and Mg-1.6Li thin films for 48 h are shown in Figure 1A. The concentrations of Mg and Mg-1.6Li in the supernatant, due to material degradation, are depicted in Figure 1B. From Figure 1A, it is clear that the degradation of Mg-1.6Li did not alter the cell proliferation of RT4-D6P2T cells when compared to the control. Mg thin films resulted in ~20% reduction in cell proliferation compared to the control (*p* < 0.01). However, this is below the cytotoxic limit of 30% proliferation reduction defined by ISO10993-5 [44]. From the Mg and Li ion concentrations shown in Figure 1B, it can be concluded that the Mg thin films degraded faster than the Mg-1.6Li thin films (*p* < 0.001), resulting in 25 mM Mg in the supernatant after 24 h. The Mg-1.6Li thin films degraded gradually, reaching a 20 mM Mg concentration only after 48 h. The Li concentration at this time point was about 1.8 mM.

### 3.2. Extracts from Freeze-Killed Cells and Nerves Triggered MCP-1 Release from RT4-D6P2T Cells

**Figure 2:**
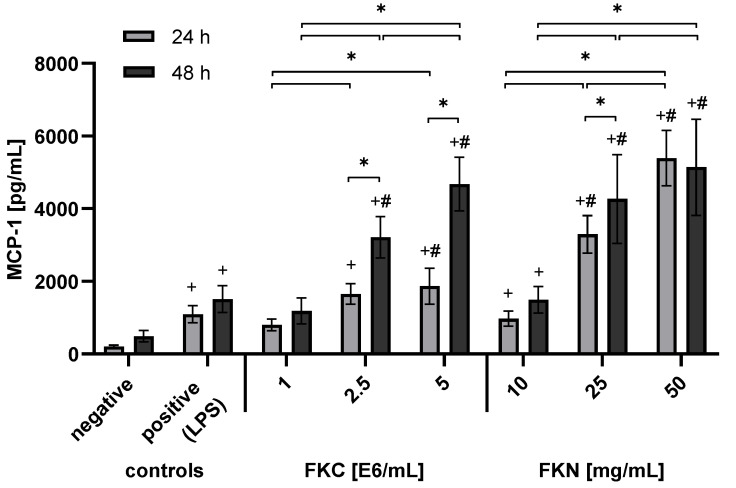
MCP-1 release induced by FKC and FKN in RT4-D6P2T cells. The concentration of MCP-1 was measured by ELISA. Untreated cells served as the negative control, and cells treated with LPS (1 µg/mL) were used as the positive control. Significant differences were identified by a two-way ANOVA (*p* < 0.001), and significance in the post hoc tests is shown for *p* < 0.05 (+, #, *). Graphs show mean ± SD of *n* = 6 data points (three replicates from two independent experiments). Symbols: +, compared to negative control; #, compared to positive control; *, comparisons between concentrations and time.

In order to mimic nerve injury in vitro, RT4-D6P2T cells were treated with FKC and FKN at varying doses, and the results of the MCP-1 ELISA measurements are summarized in Figure 2. Both FKC and FKN induced a dose-dependent increase in MCP-1 release. FKN (50 mg/mL) induced the highest MCP-1 release, up to ~6000 pg/mL at 24 h. The highest MCP-1 released by FKC (5 × 10^6^ cells/mL) was ~2000 pg/mL at 24 h, and this amount increased by 2.3-fold at 48 h (*p* < 0.05). At higher doses, both FKC and FKN were more effective than LPS in inducing MCP-1 release (*p* < 0.05). To meet the requirement for larger quantities of extracts for further experiments, FKC (5 × 10^6^ cells/mL) was chosen for practicality and to conserve animal use. 

### 3.3. Influence of Mg-Based Thin Films on RT4-D6P2T Cellular Response to Injury

RT4-D6P2T cells were treated with FKC extract (5 × 10^6^ cells/mL) to simulate a nerve injury-like environment in vitro, and the effects of the Mg/Mg-1.6Li thin films on various cellular responses were studied over a period of 48 h. 

#### 3.3.1. Mg/Mg-1.6Li Thin Films Reduced MCP-1 Release from FKC-Treated RT4-D6P2T Cells

**Figure 3:**
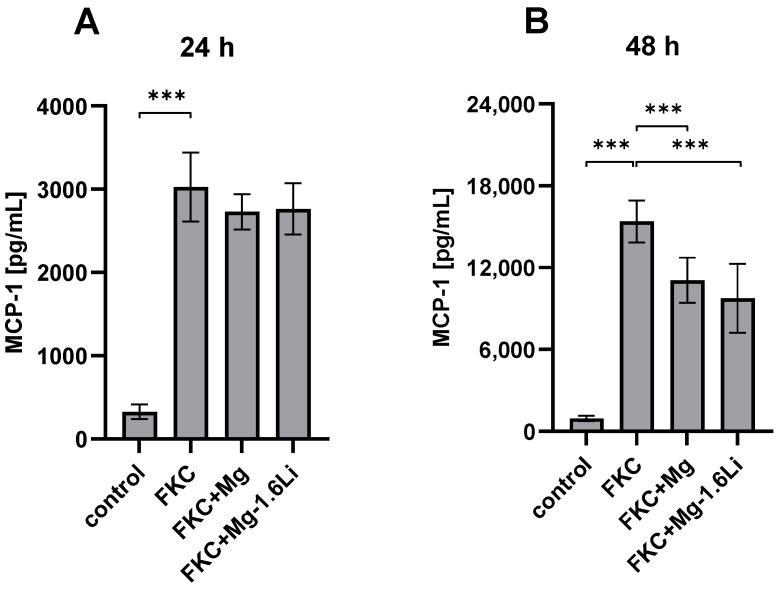
Effect of Mg and Mg-1.6Li thin film degradation on MCP-1 release induced by FKC in RT4-D6P2T cells. The concentration of MCP-1 was measured by ELISA. (**A**) After 24 h; (**B**) After 48 h. Significant differences were analyzed by one-way ANOVAs (*p* < 0.001, each for 24 and 48 h), with post hoc test significance levels indicated at *p* < 0.001 (***). Graphs show mean ± SD of *n* = 9 data points (three replicates from three independent experiments).

The effect of the thin films on MCP-1 release is shown in Figure 3. After 24 h of exposure, the thin films had no significant effect on MCP-1 release (Figure 3A). However, after 48 h, both thin films significantly reduced MCP-1 release by ~30% (Figure 3B, *p* < 0.001). Although the Mg concentration at 24 h due to the effect of the Mg thin films was higher than that with Mg-1.6Li thin films (Figure 1B), this did not influence the MCP-1 release, thereby suggesting a time-dependent effect that was not related to the Mg concentration.

#### 3.3.2. The Gene Expression of Neurotrophins Is Regulated Differently by the Thin Films in the Presence of the Injury Stimulant

**Figure 4:**
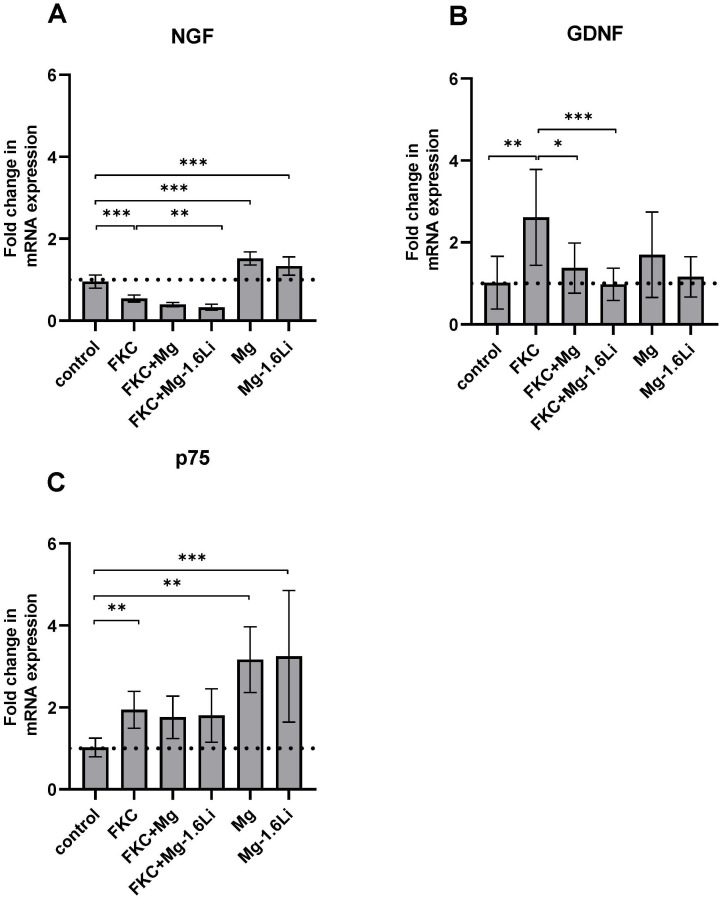
Effect of Mg and Mg-1.6Li thin film degradation on the expression of neurotrophin-related genes as measured by RT-qPCR. (**A**) NGF; (**B**) GDNF; (**C**) p75. Graphs show mean ± SD of *n* = 5–9 data points (three replicates from three independent experiments, outliers excluded). The dotted line represents the control. Significant differences were identified by one-way ANOVAs (*p* < 0.001 for each gene), and significance in the post hoc tests is indicated at *p* < 0.05 (*); *p* < 0.01 (**); *p* < 0.001 (***).

The gene expression of two neurotrophins, NGF and GDNF, and the pan neurotrophin receptor p75 were analyzed by RT-qPCR after 48 h of indirect exposure to thin films, and the results are shown in Figure 4. Cells treated with FKC showed a significantly reduced gene expression of NGF compared to the control (Figure 4A, post hoc test *p* < 0.001). Interestingly, the cells also exposed to Mg-1.6Li thin films (FKC+Mg-1.6Li) showed a slightly additional downregulation of NGF expression (*p* < 0.01). In the absence of FKC, both thin films upregulated the gene expression of NGF when compared to that of the control (*p* < 0.001). The gene expression of GDNF was significantly upregulated due to FKC (Figure 4B, *p* < 0.01), and the addition of thin films (FKC+Mg, FKC+Mg-1.6Li) downregulated the gene expression level compared to that of the FKC-treated cells (*p* < 0.05). In the absence of FKC, the thin film degradation did not significantly influence the GDNF gene expression. The p75 gene expression was upregulated two-fold due to FKC stimulation (Figure 4C, *p* < 0.01 vs. control) and this increase remained uninfluenced by the thin films. However, in the absence of FKC, the thin films upregulated the gene expression of p75 up to three-fold when compared to that of the control (*p* < 0.01).

#### 3.3.3. Mg/Mg-1.6Li Thin Films Influenced the Expression of Key Myelin Protein Genes

**Figure 5:**
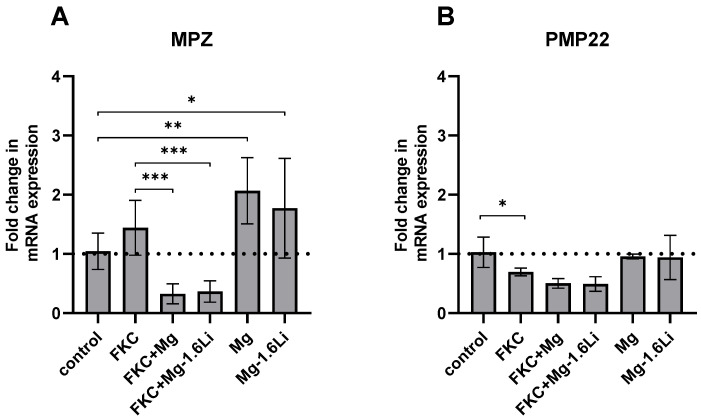
Effect of Mg and Mg-1.6Li thin film degradation on the expression of myelin protein genes as measured by RT-qPCR. (**A**) MPZ; (**B**) PMP22. Graphs show mean ± SD of *n* = 5–9 data points (three replicates from three independent experiments, outliers excluded). The dotted line represents the control. Significant differences were identified by a one-way ANOVA (*p* < 0.001 for each gene), and significance in the post hoc test is indicated at *p* < 0.05 (*); *p* < 0.01 (**); *p* < 0.001 (***).

Two myelin proteins, MPZ (myelin protein zero) and PMP22 (peripheral myelin protein 22), which are known to be expressed by RT4-D6P2T cells [45], were analyzed, and the results are presented in Figure 5. The gene expression of MPZ was not altered by the presence of FKC alone but was downregulated by the thin films in the presence of FKC (*p* < 0.001). In the absence of FKC, the gene expression of MPZ increased up to two-fold in the presence Mg (*p* < 0.01) and Mg-1.6Li (*p* < 0.05) when compared to the control (Figure 5A). The gene expression of PMP22 was slightly downregulated due to FKC (Figure 5B, *p* < 0.05), and the thin films showed no further effects on the expression of this gene.

## 4. Discussion

Improved treatment strategies for peripheral nerve injuries have been a focal point of research in regenerative medicine [1]. Recent research suggests that Mg-based materials can provide both trophic support [15,17], as well as bioactive, pro-regenerative contact guidance for nerve regeneration [20,21]. However, there is a lack of detailed understanding about the effects of Mg degradation on SC responses. Therefore, we used an in vitro injury model employing the RT4-D6P2T Schwannoma cell line and tested the effects of Mg and Mg-1.6Li alloy degradation on various injury-relevant responses. Although it is challenging to reproduce the complexity of peripheral nerve injury in vitro, cell lines can be advantageous to study specific responses by reducing variability, as well as animal use [46,47]. Moreover, isolation of primary SCs can be challenging due to fibroblast contamination [48,49]. Therefore, it is beneficial to develop and understand cell line-based models for fundamental research in peripheral nerve regeneration. 

In this study, we cultured RT4-D6P2T cells and provided indirect contact with Mg and Mg-1.6Li thin films across an insert membrane. This not only exposed the cells to the ionic products from continuous material degradation, but also to a dynamic pH environment and transitory changes such as hydrogen gas release [50]. In this case, even though both Mg and Mg-1.6Li resulted in similar Mg concentrations after 48 h, the Mg thin films degraded faster. Cells can endure a gradual change in osmolality better than sudden osmotic stress [51]. The more abrupt increase in osmolality and/or rapid increase in Mg^2+^ concentration due to Mg thin film degradation could explain the mild adverse effect on cell proliferation. The degradation of Mg-1.6Li thin films was more gradual, without any effect on the cell proliferation. Either way, 20–25 mM Mg was not significantly toxic to these cells. The degradation of Mg-1.6Li resulted in Li concentrations slightly above the serum therapeutic range for humans (0.6–1.2 mM) [38], with no significant effects on cell proliferation, which agrees with the results of other in vitro studies [34,52,53]. For future applications, long term degradation and cytotoxicity studies are essential, and the effects of varying Mg and Li concentrations must be tested. These changes will require adjustments in the assay conditions and material degradation rates. The degradation rate can be tailored to achieve the effects most conducive to nerve regeneration through multiple means, including surface modification, e.g., coatings [54], or material optimization using in silico approaches [55,56].

In order to create a microenvironment resembling that after nerve injury, we used extracts derived from freeze-killed cells and nerves. Upon nerve injury, damage-associated molecular patterns (DAMPs) are released from necrotic dead cells, which activate the innate immune response. DAMPs include TLR ligands, such as heat shock proteins, extracellular matrix components, nucleic acids, and purinoreceptor ligands, such as nucleotides [42]. Accordingly, we found that both FKC and FKN triggered an inflammatory response in RT4-D6P2T cells, marked by an increased MCP-1 release. Such a response due to FKC and FKN was previously demonstrated only in primary SCs [40,41]. While FKC showed a more time-dependent increase in MCP-1 release, the FKN-induced MCP-1 release seemed to already be around or at the dose-dependent 48 h maximum at 24 h. The extent of MCP-1 release due to FKC and FKN cannot be directly compared, as no common parameter was used to normalize the doses of the two stimulants. However, a qualitative difference in the pattern of the MCP-1 release was observed. FKN contains additional factors such as axonal debris, myelin proteins, and lipids, as well as necrotic factors from the vasculature and surrounding fibroblasts [4]. Although Karanth et al. stated that myelin proteins were ineffective in inducing MCP-1 release [41], Xu et al. showed that saturated fatty acids, which are abundant in myelin, activated an inflammatory response in SCs via TLR signaling [57,58]. In summary, both FKC and FKN can serve as complex in vitro homologues to nerve injury. 

The influence of FKC on RT4-D6P2T cells was also evident in the gene expression analysis, which revealed an upregulation in the expression of GDNF and p75. The transcription factor c-Jun is a master regulator of the SC transformation to the repair phenotype [59]. GDNF is upregulated after nerve injury, mainly in a c-Jun-dependent manner, and this growth factor is important for neuronal survival and axonal regeneration [60]. The p75 is a neurotrophin receptor that is also upregulated after injury and is considered as a marker of SC dedifferentiation [59,61]. It plays an important role in functional recovery [62,63]. NGF has a protective role [63], similar to that of GDNF. However, contrary to expectations [4], NGF was slightly downregulated in the cells exposed to FKC. In terms of myelin proteins, the repair phenotype of SCs is known to downregulate their production [58]. In our study, only the gene expression of PMP22 was significantly downregulated due to FKC. In conclusion, RT4-D6P2T cells treated with FKC show some responses similar to those of SCs after nerve injury but not all aspects of the repair phenotype observed in vivo. Nonetheless, this study shows that necrotic cell extracts can be used as a reproducible in vitro injury model using a Schwann cell line. 

In the next step, the RT4-D6P2T cells, with or without FKC, were exposed to Mg and Mg-1.6Li thin films. The thin films led to a significant drop in MCP-1 release, implying a reduced pro-inflammatory response in the presence of thin film degradation. This observation conforms to the known anti-inflammatory effects of both Mg and Li via the NF-κB pathway [64,65,66]. MCP-1 is an important chemokine for macrophage recruitment after nerve injury [4]; therefore, a reduction in macrophage-driven myelin phagocytosis and inflammation could be expected. However, the reduced inflammation could be beneficial to healing [14,67]. Moreover, studies have shown that macrophage recruitment can occur independent of MCP-1 [68], and SCs are also capable of degrading myelin [69,70].

The gene expression of NGF and GDNF in FKC-treated cells was reduced by the thin films. As the secretion of these growth factors is primarily c-Jun dependent, these results suggest that Mg and Li can affect c-Jun upregulation after nerve injury. Different groups have shown that Li reduces the c-Jun stress response in both neurons [71,72] and SCs [53]. Also, c-Jun levels have an inverse relationship with Mg concentrations [73,74], but this has not been shown in the context of nerve injury. Further experiments are necessary to understand the regulation of the c-Jun pathway by Mg degradation products after nerve injury. Interestingly, the increased expression of p75 due to FKC was unaffected by the thin film degradation, suggesting that this upregulation is governed by another, independent pathway. A reduction in c-Jun, but maintenance of p75 levels, has been observed previously [53,60]. In our study, this is likely due to the ERK (extracellular signal-regulated kinase)-activating effect of Mg [75,76,77], which is another key pathway known to affect SC plasticity [5]. 

**Figure 6:**
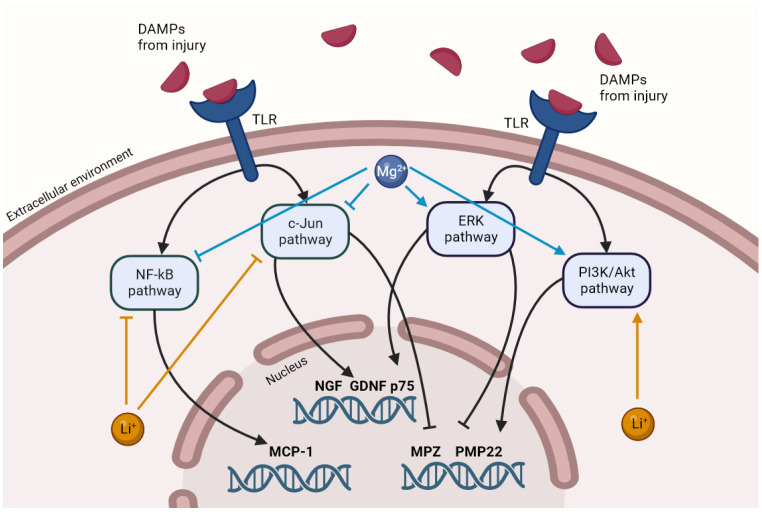
The possible mechanisms of action of Mg and Li on the repair pathways in injury stimulated SCs, as inferred from the literature. Only the mechanisms relevant to the observations in this study are depicted.

Both Mg and Li are known to enhance remyelination [15,16,30,35], mainly due to their upregulation of the PI3K/Akt (phosphoinositide 3-kinase/protein kinase B) pathway [5,34,77]. However, a remarkable observation in our study was that both thin films reduced myelin gene expression in the presence of FKC, suggesting that a different mechanism is at play. Ogata et al. showed how the PI3K/Akt and ERK pathways have opposing effects on SCs, and their balance determines the expression of myelin genes [34]. The ERK pathway plays a crucial role in SC dedifferentiation after injury [5], and Mg further upregulates this enzyme. In addition, the activation of c-Jun after injury also downregulates myelin protein production [5]. The combination of these effects could lead to the observed downregulation of myelin protein genes in the presence of both FKC and Mg. Further studies are necessary to compare the extent of PI3K/Akt and ERK activation in SCs due to Mg degradation. The previously known effects of Mg and Li on the pathways governing the repair phenotype in SCs are summarized in Figure 6.

In the absence of FKC, both thin films upregulated the expression of NGF, p75, and MPZ, hinting that Mg-based materials may improve nerve regeneration during the later stages of healing when DAMPs are absent. A similar increase in NGF expression due to Mg-based materials has been shown previously [17,19,23]. A significant effect due to alloying Li at 1.6wt% could not be identified with any of the tested parameters used in our study. Mg-driven effects were probably prominent due to the concentration difference, and higher Li concentrations might be necessary to observe its effects in the presence of Mg. Overall, RT4-D6P2T responses to Mg degradation revealed wide-ranging and sometimes contrasting effects in the acute phase. Nevertheless, the results support the observations from many in vivo studies that Mg promotes different aspects of nerve regeneration [14,15,20].

Of particular interest in our findings was that the Mg degradation products had very different and in some cases, opposite effects on gene expression in SCs, depending on the exposure to injured cell materials. The bioabsorbable metals like Mg have been challenging to study in vitro, although the main focus has been on the fact that the metal degradation rates in vitro do not match those in vivo, and that Mg degradation in biological fluids is very complex [78,79]. This lack of optimal in vitro models has hampered the development of bioabsorbable metal implants for biomedical purposes, specifically by requiring adjustments in the standards and guidelines for the pre-clinical analysis of bioabsorbable metals [80]. Our results demonstrate that another factor, cellular changes after exposure to an injury, must be considered in characterizing the effectiveness and functional impact of tissues adjacent to Mg metal implants and the interpretation of in vitro studies. Here, we show one approach of applying one type of in vitro injury model to absorbable metal analyses, but further work is needed to refine the conditions and explore the underlying mechanisms, thereby advancing studies of the benefits of biodegradable metals for the regeneration of nerves and other tissues.

## 5. Conclusions

In this study, we used an in vitro model to test the effects of Mg and Mg-1.6Li material degradation on acute injury-related responses of RT4-D6P2T Schwannoma cells. Both freeze-killed cells and nerve extracts induced injury-related responses from SCs, and the results suggest that additional factors in nerve debris could play a role in the responses. The SC changes in response to the freeze-killed cell extracts were reminiscent of the post-injury, repair SC phenotype, including the increased release of MCP-1, and with cell extracts, changes in the gene expression of neurotrophins and myelin proteins. Thus, this method shows promise as a model of treatment for in vitro nerve injury. The degradation products of Mg/Mg-1.6Li reduced the injury-induced SC release of MCP-1, which is consistent with the known anti-inflammatory effects of Mg, and altered several key SC gene expression responses to injury. Notably, SC changes were different in the absence of injury materials. We did not observe any additional effects due to the small amount of Li released by the Mg-1.6Li alloy, suggesting that higher Li concentrations may be necessary for an observable difference. Overall, these results demonstrate that cellular changes due to the microenvironment in a wound site after injury, modeled in vitro, altered cellular responses to a bioabsorbable metal, Mg. Therefore, in vitro models of nerve injury like the ones we explored here show promise for studies of the beneficial effects and wide-ranging mechanisms of Mg in supporting nerve regeneration specifically and tissue regeneration in general. 

## Figures and Tables

**Table 1:** List of primer pairs used for qPCR.

Gene	Primer Sequence
*GAPDH* (reference gene)	Forward GGCAAGTTCAACGGCACAG Reverse CGCCAGTAGACTCCACGAC
*NGF*	Forward AGCTCACCTCAGTGTCTGG Reverse GCTATCTGTGTACGGTTCTGC
*GDNF*	Forward TCGGGCCACTTGGAGTTAAT Reverse CAGCCACGACATCCCATAAC
*p75*	Forward CAACCAGACCGTGTGTGAACC Reverse GTCTCCTCGTCCTGGTAGTAGC
*MPZ*	Forward CACCACTCAGTTCCTTGTCC Reverse ACTTCCCTGTCCGTGTAAACC
*PMP22*	Forward TGTACCACATCCGCCTTGG Reverse CTCATCACACACAGACCAGCAAG

## Data Availability

The relevant data are provided in the manuscript, and any further data that support the observations in this study can be provided by the corresponding author upon reasonable request.
